# Unveiling unique expression patterns of D20S16 satellite DNA in human embryonic development

**DOI:** 10.1038/s41598-025-11753-w

**Published:** 2025-07-23

**Authors:** Yajie Hu, Kenji Mizuguchi, Kosuke Hashimoto

**Affiliations:** Institute for Protein Research, Osaka University, Osaka, Japan

**Keywords:** Satellite DNA, D20S16, Embryonic development, RNA-seq, Comparative genomics, Human genome (T2T-CHM13), Gene expression, Computational biology and bioinformatics, Embryogenesis

## Abstract

Satellite DNA is essential for chromosome stability and gene regulation, yet its specific roles in early human embryogenesis remain poorly defined. Here, we integrated the complete human genome reference (T2T-CHM13) with RNA-seq data to investigate the expression and regulation of the satellite DNA element D20S16 across key stages of human embryonic development. We identified 20 distinct D20S16 tandem repeat clusters, but found that only two, both located on chromosome 20, were actively transcribed during early embryogenesis. Expression of D20S16 was high in early developmental stages, significantly declining thereafter. Comparative analysis revealed minimal expression of D20S16 in macaque embryos, correlating with fewer and shorter repeat units. Beyond embryogenesis, D20S16 also exhibited notably high expression levels in breast cancer and testicular tissues, suggesting additional biological roles. Furthermore, we investigated the evolutionary distribution of D20S16 across primates and other mammals. Our findings highlight the potential regulatory functions of satellite DNA in human development, pointing to the importance of specific chromosomal contexts for transcriptional activation. This study enhances our understanding of satellite DNA’s functional and evolutionary significance, laying the groundwork for future research into its roles in development and disease.

## Introduction

Embryonic development, a pivotal stage in mammalian life, begins with the fusion of sperm and egg. This marks the start of a major transformation involving chromatin structure and transcriptional activity. The process starts with a totipotent fertilized egg and in humans proceeds through cleavage, morula, and blastocyst stages. This succession of early embryonic events essentially represents a process of cell proliferation with large-scale epigenetic remodelling, involving various biological processes that require precise transcriptional regulation, epigenetic reprogramming, and orderly metabolic changes^1^.

Recent advances in RNA sequencing technologies have deepened our understanding of transcriptional changes in embryos at different stages^2,3^. Epigenetic reprogramming during gametogenesis and embryonic development has also been progressively revealed^4–6^. However, despite these advancements, knowledge of satellite DNA expression during human embryonic development remains limited.

A substantial portion of the genome is non-coding DNA, including tandem repeat sequences such as satellite DNA that occupy many eukaryotic chromosomes. These sequences are predominantly located in (peri)centromeric and (sub)telomeric regions of chromosomes. Satellite DNA refers to tandemly repeated sequences broadly classified by repeat unit length into microsatellites (1–6 bp), minisatellites (10–100 bp), and classical satellite DNA (> 100 bp, often megabase-scale arrays in centromeric regions)^7^, and while predominantly found in non-coding regions, they can also be present within or adjacent to protein-coding regions, potentially influencing transcriptional regulation, protein structure, or genomic stability^8^. Initially, owing to their non-coding nature and lack of sequence conservation across closely related species, satellite DNAs were primarily considered “junk DNA”^9^. However, contemporary research has revealed that satellite DNA and their transcripts play critical roles in chromosomal segregation, genome stability, and early embryonic development by regulating heterochromatin organization and chromocenter formation^10–18^. Therefore, a thorough investigation into the role of satellite DNA in embryonic development is essential for unveiling the intricate mysteries of embryogenesis.

In the past, the study of satellite DNA was particularly challenging owing to their short repetitive sequences. In addition, the incompleteness of the human GRCh38 reference genome, with missing or incorrect data in over 5% of sequences^19–21^, compounded the difficulty. These missing or incorrect parts were located mainly where satellite DNA is prevalent, notably in telomeric and centromeric regions. Moreover, the classification and detection of satellite DNA is further complicated by its structural complexity and potential sequence divergence among repeat units^8^. However, with newer DNA sequencing technologies, such as PacBio HiFi and Oxford Nanopore’s ultra-long-read sequencing^22^, the Telomere-to-Telomere (T2T) Consortium successfully filled these regions and released the first complete human genome reference, CHM13^23^. Furthermore, it has completed the sequence information for the Y chromosome, unveiling v. 2.0 This breakthrough provides invaluable data for our research into satellite DNA.

This article presents our findings on satellite DNA expression during human embryogenesis, specifically focusing on D20S16 due to its exceptionally high expression. Although previously annotated as satellite DNA, D20S16 is composed of short tandemly arranged units (49–98 bp) and resides within a genic context, characteristics that more closely align with minisatellite DNA rather than classical centromeric satellites^7^. Investigating D20S16 provides insights into satellite DNA regulation and its potential biological functions. A previous study has demonstrated that D20S16 is expressed during early human embryogenesis, peaking specifically at the 4-cell stage^24^, highlighting its possible involvement in critical processes such as chromatin remodeling and embryonic genome activation.

## Results

### Stage-specific expression patterns of D20S16

**Fig. 1:**
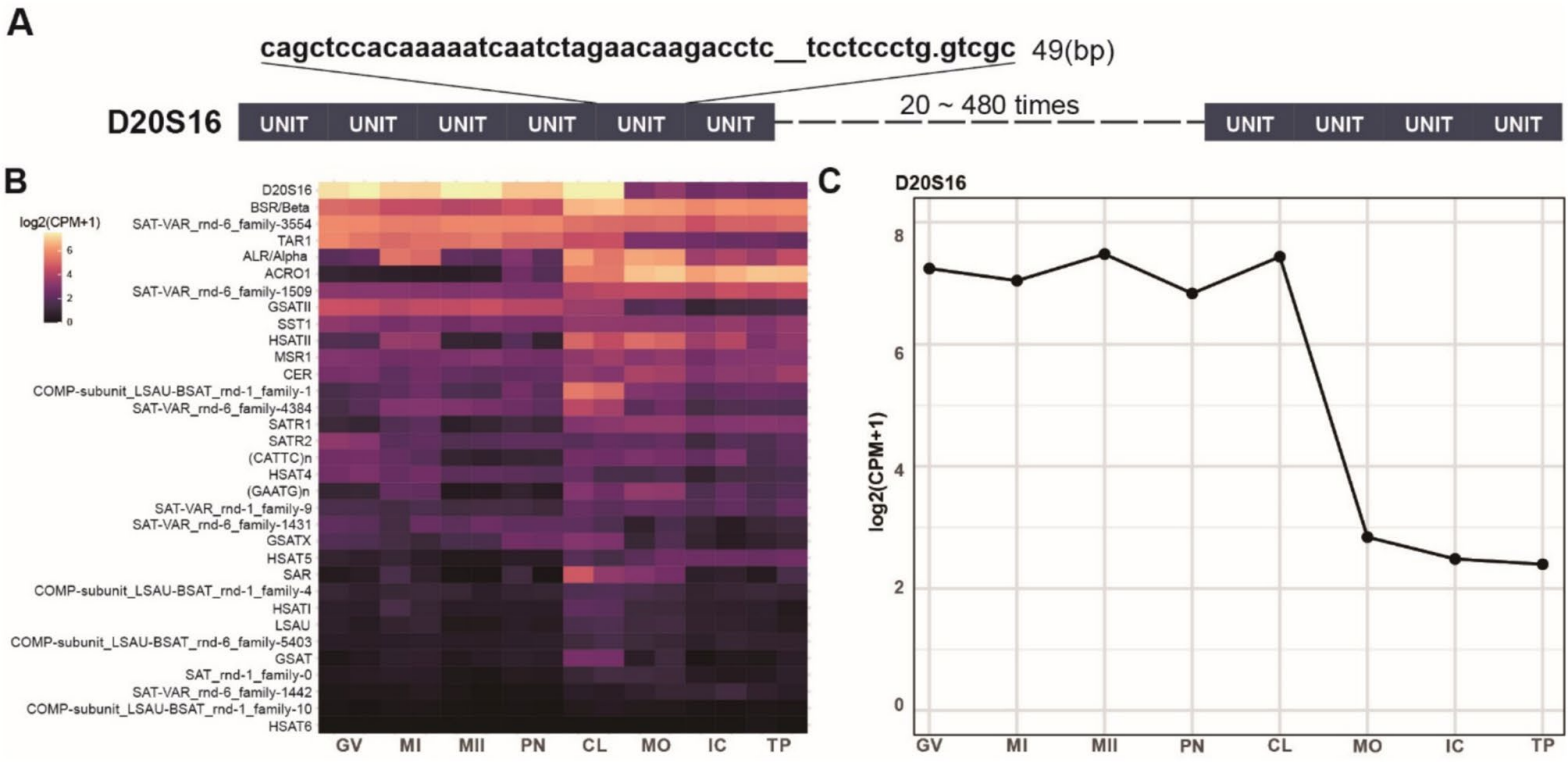
Satellite DNA expression in embryonic development. (**A**) Schematic representation of the D20S16 locus, showcasing a single-copy sequence composed of sequential-dynamic-variability repetitive units (49 bp). (**B**) Heatmap illustrating expression patterns of 33 satellite DNAs across eight stages of human embryonic development: Germinal Vesicle (GV), Meiosis I (MI), Meiosis II (MII), Pronuclei (PN), Cleavage (CL), Morula (MO), Inner Cell Mass (IC), and Trophectoderm (TP). CPM = counts per million. To visualize gene expression levels, we normalized data as log_2_(CPM + 1). (**C**) D20S16 expression is high before CL (cleavage) stage and decreases after.

Satellite DNA, comprising highly repetitive non-coding sequences, has traditionally been categorized into distinct types based on sequence features and chromosomal localization. While RepeatMasker annotations have reported approximately 33 types in the human genome, recent systematic analyses suggest a much broader diversity of satellite families, reflecting ongoing discoveries in satellite DNA classification and evolution^7^. Among these satellite DNA types, D20S16 has been characterized as a complex interspersed repeated sequence on chromosome 20q12-q13.1^25^. It comprises variable numbers of tandemly repeated 98-bp units, each consisting of two highly similar 49-bp subunits (87–90% identity) (Fig. 1A). The polymorphism observed at D20S16 mainly arises from differences in the number of these repeat units.

We analysed an RNA-Seq dataset^26^ (SRA ID SRP062850) representing a comprehensive set of transcriptomes of human oocytes and preimplantation embryonic development. This dataset includes samples from seven stages of human oogenesis and early embryogenesis, processed with a transposase-based library method to sequence total RNA without 3′ bias^26^. Through analysis, we have discovered that satellite DNAs exhibit stage-specific expression patterns during embryonic development (Fig. 1B). D20S16, along with GSATII and TAR1, shows high expression before the cleavage (CL) stage of embryonic development, followed by a noticeable decrease in later stages (Fig. 1C). Conversely, other satellite DNAs, such as ACRO1^27^ and BSR/Beta, display increasing expression after the CL stage.

To validate these findings and enhance the robustness of our observations, we used an additional dataset^28^ (SRA ID SRP061636), which encompasses a comprehensive set of transcriptomes, covering both polyA + and polyA − mRNAs. The results corroborated our initial observations and provided further insights into the timing of D20S16 expression decline. Specifically, while the first RNA-Seq analysis showed a marked decrease in D20S16 expression after the CL stage, the second dataset showed that this decline begins after the 8-cell (8C) stage, which is part of the cleavage stage (Fig. S1). Furthermore, the 2-cell (2C), 4-cell (4C), and 8-cell (8C) stages included in this dataset provide more detailed insights into the expression dynamics of satellite DNA during this critical developmental phase. These results demonstrate that satellite DNAs, such as D20S16, exhibit distinct expression patterns during embryonic development.

### Discovery of twenty distinct yet continuous D20S16 elements in the human genome

**Fig. 2:**
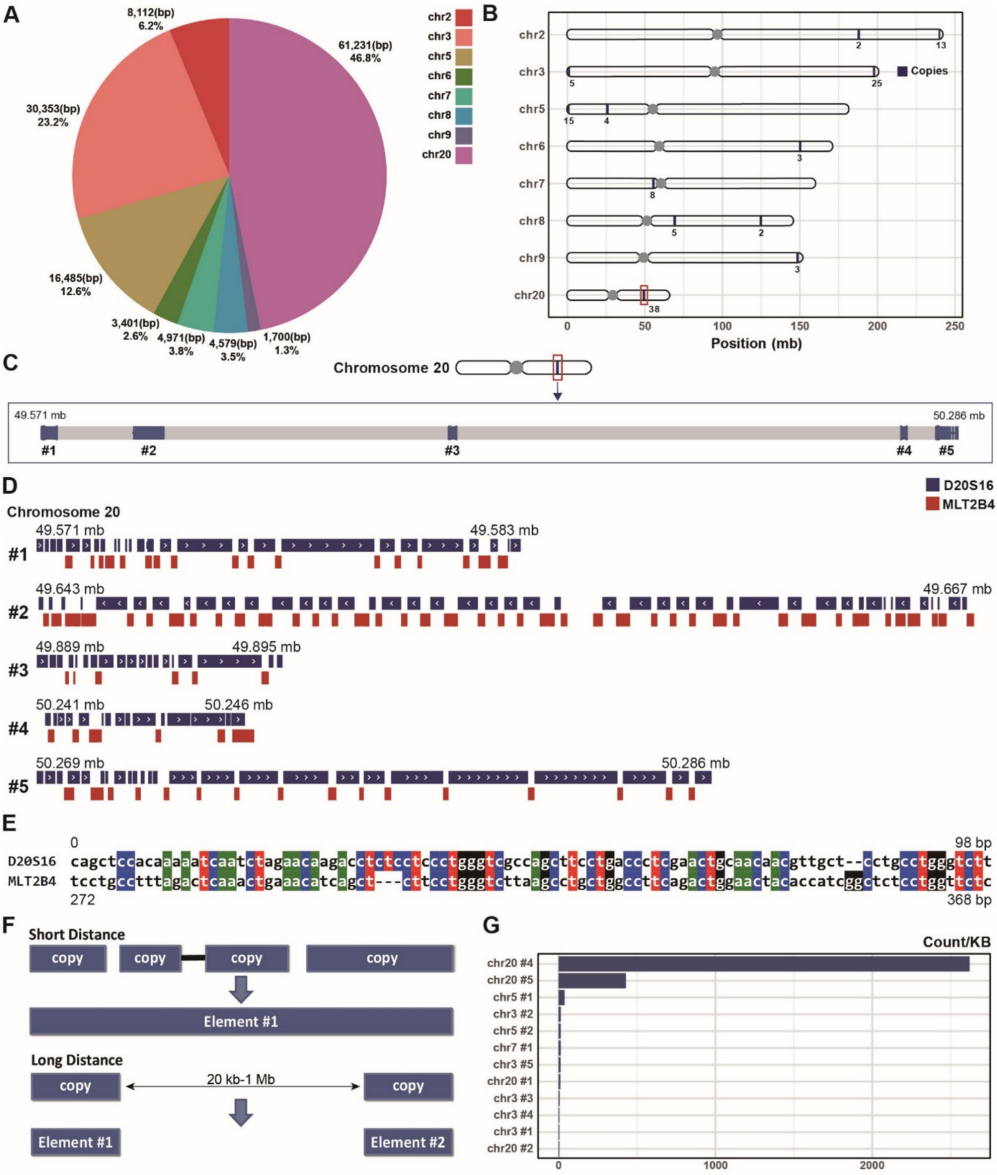
D20S16 distribution in the genome. (**A**) Number and total base-pair length of D20S16 elements per chromosome. Chr. 20 contains 61,231 bp of D20S16 sequence, which accounts for 46.8% of the total D20S16 length across the genome. **(B)** The distribution of D20S16 copies across different chromosomes. Gray bar shows the length of each chromosome. Dark blue points mean each copy site. Boxed point means 38 copies clustered at the 50 Mb region on chr. 20. **(C)** Map outlining the specific locations of D20S16 sequences on chr. 20 from 49.571 to 50.286 Mb, numbered from #1. **(D)** Comparative genomic map displaying the relative positions of D20S16 and MLT2B4 on the T2T-CHM13 chr. 20. Insertion of MLT2B4 into D20S16 leads to a RepeatMasker annotation error and misidentification of D20S16 regions. **(E)** Alignment comparison highlighting sequence similarities between MLT2B4 and D20S16: part of MLT2B4 (272–368 bp) has similarity to D20S16. **(F)** The process of integrating individual copies of D20S16 into composite elements, we categorized the distances between the copies on each chromosome, using the copies with larger intervals as demarcation points and combining copies with smaller intervals into a single, continuous D20S16 region. **(G)** D20S16 copy expression levels on different chromosomal elements. Expression in chr20#4 and #5 is much higher than in the other elements.

After identifying the stage-specific expression patterns of D20S16, the next challenge was to determine which of the many D20S16 copies in the genome were actually being transcribed. To address this, we first needed to clarify the exact number and locations of D20S16 copies in the human genome. Using RepeatMasker annotations, we initially identified 123 copies of D20S16 dispersed throughout the human genome (Fig. S2), 38 on chromosome (chr.) 20 and 30 on chr. 3, and the rest distributed across chrs. 2 and 5 to 9. On chr. 20, D20S16 spans 61,231 base pairs (bp), accounting for 46.8% of the total D20S16 sequence length identified across all chromosomes (Fig. 2A). We found a marked tendency to cluster in specific regions (Fig. 2B): specifically, 38 copies on chr. 20 are entirely concentrated into 5 clusters around the 50 Mb region, each separated by very short distances (Fig. 2C).

Upon closer examination using the Integrative Genomics Viewer (IGV)^29^, we realized that these clustered D20S16 were not isolated copies but instead components of a continuous genomic region, forming a single long D20S16 element composed of tandemly arranged repetitive units (Fig. 2D). These segments were mistakenly annotated as MLT2B4 due to sequence similarities between parts of D20S16 and MLT2B4 (Fig. 2E). While the consensus sequence of MLT2B4 is 557 bp, full-length LTR retrotransposons typically span several kilobases, suggesting that these ~ 100 bp fragments are incomplete and likely represent misannotated segments rather than functional retrotransposon insertions (Fig. 2D). Sequence comparison revealed that a part of MLT2B4 (272–368 bp) has similarity to D20S16 (Fig. 2E), which led to these mis-annotations.

Expression data displayed continuous transcription from a broad region of D20S16, indicating that the repetitive units—although annotated as separate copies and occasionally interrupted by short sequences labeled as “MLT2B4”—are in fact transcribed as a single, continuous unit. This observation supports the view that these regions constitute a single, larger D20S16 tandem repeat cluster, rather than a mixture of independent D20S16 and retroelement transcripts. To better reflect this structural continuity, we consolidated the 123 RepeatMasker-annotated D20S16 units—many of which were fragmented by short MLT2B4-like insertions—into 20 distinct tandem repeat clusters. For clarity, we refer to these clusters as “elements” throughout the remainder of the manuscript (Fig. 2F; Table S1). This reannotation substantially altered the perceived sequence architecture, transforming multiple short and discontinuous annotations into fewer, but much longer, consolidated elements (Fig. S3).

The expression analysis of the 20 refined elements revealed that two adjacent elements on chr. 20 (chr20 #4 and chr20 #5) are predominantly expressed in total of GV-CL (Fig. 2G). These two elements accounted for the vast majority of D20S16 expression in embryo. In contrast, elements on chrs 3, 5 and 7 exhibited minimal expression and no expression was detected from other chromosomes, as confirmed by the second dataset (Fig. S4).

### Identifying D20S16 repeat units via manual extraction and hidden markov models

**Fig. 3:**
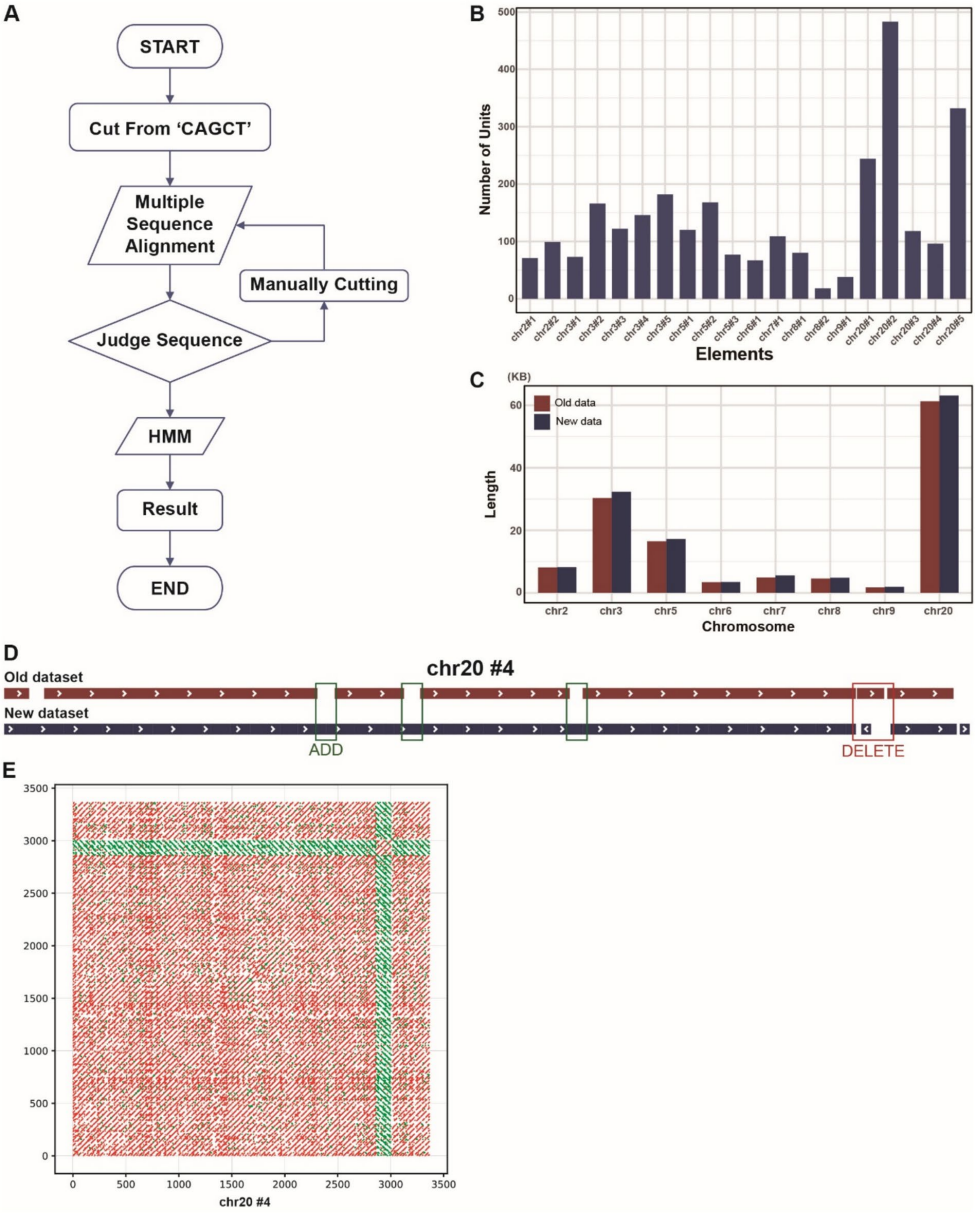
The composition of D20S16, along with the revised workflow and results. (**A**) Diagram outlining the step-by-step protocol for isolating D20S16 repetitive units. We manually investigated and identified a conserved 5-amino-acid motif, “CAGCT”, in the repetitive units. Consequently, we resorted to manual extraction, using “CAGCT” as a reference point for cutting and aligning multiple sequences for all 20 refined elements. We further corrected and adjusted sequences with minor variations, including “CAGCC” or “CATGT”. After 2 or 3 iterations of this process, we used Hidden Markov Models (HMMs) to capture repetitive units potentially missed during manual extraction. **(B)** Bar graph quantifying repetitive units identified within each D20S16 element: 483 were located in chr20#2, 332 in chr20#5, and 96 in chr20#4. **(C)** Describes the differences in base lengths between the RepeatMasker data (raw data) versus newly extracted data (new data). **(D)** A comparison of the old and new D20S16 using chr20 #4 as an example revealed the addition of regions previously misannotated, as well as the removal of redundant insertions with errors. **(E)** Self-alignment dot plot of the full-length chr20#4 sequence, revealing extensive internal similarity and supporting the presence of a high-order tandem repeat structure. Red lines represent forward strand alignments; green lines indicate reverse complement matches.

To elucidate the reasons behind the specific high expression of D20S16 from chr20#4 and chr20#5, we aimed to explore the internal sequence composition of these elements. D20S16 is composed of short repetitive units, each 49 bp long^25^ (Fig. 1A). However, whether the sequences of the repetitive units vary among different copies remains unclear. We tried to extract these repetitive units from the chr20#4 and #5 elements using publicly available tools such as RepeatModeler2^30^ and RepEx^31^, but failed owing to their high diversity and short sequence lengths. We then manually investigated and identified a highly conserved 5-nucleotide motif, “CAGCT”, in the repetitive units. Consequently, we resorted to manual extraction, using “CAGCT” as a reference point for cutting and aligning multiple sequences from all 20 refined elements. We further corrected and adjusted sequences with minor variations, including “CAGCC” or “CATGT”. After 2 or 3 iterations of this process, we extracted 2611 repetitive units from the 20 elements, and generated a single multiple sequence alignment (Fig. 3A).

Using the multiple sequence alignment, we used Hidden Markov Models (HMMs)^32^ to capture repetitive units potentially missed during manual extraction. We built an HMM model using the *hmmbuild* function in HMMER (v. 3.3.2) and searched for repetitive units in the human genome using the *hmmsearch* function (E-value < 0.01).

Finally, we identified 2809 repetitive units (Fig. 3B) with an average length of 53 bp. Of these, 483 were located in chr20#2, 332 in chr20#5, and 96 in chr20#4. Despite their relatively smaller number, the repetitive units in chr20#4 had remarkably high expression levels. To further explore the internal organization of chr20#4, we performed sequence self-alignment and dot plot analysis. The results revealed a tandem repeat architecture consistent with a highly ordered repeat cluster structure (Fig. 3E). Compared with the RepeatMasker data (Fig. 3C), our approach added 8528 bases and removed 2811 misannotated bases (Fig. 3D), refining the D20S16 profile and establishing a new HMM model for further research.

### Identifying four types of D20S16 on the basis of the variable region

**Fig. 4:**
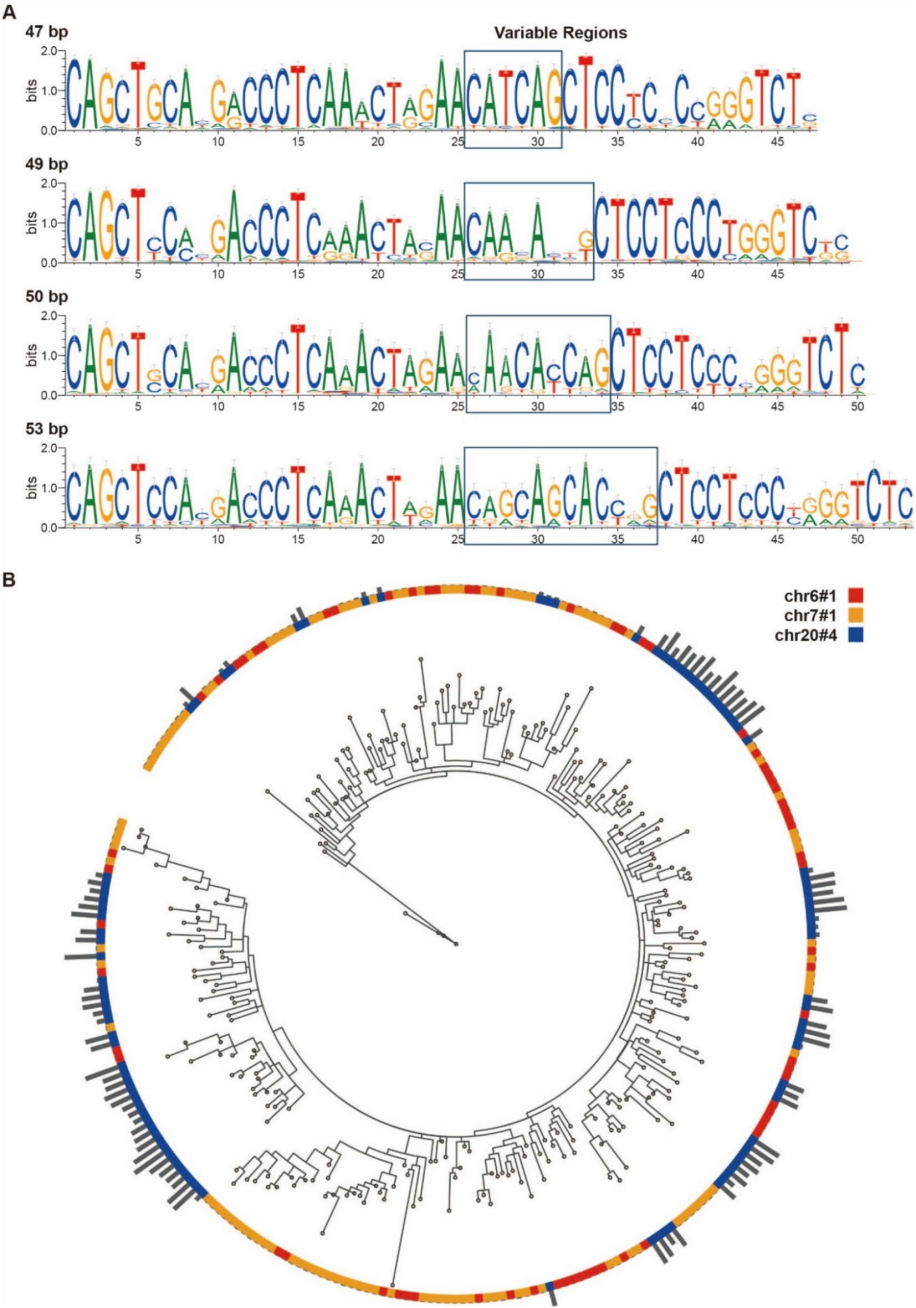
The consensus sequence and phylogenetic tree of D20S16. (**A**) Sequence logos illustrating the consensus sequence and variable regions for D20S16 units from the 47-, 49-, 50- and 53-bp groups. Larger letters mean they’re more conservative in their position. **(B)** Phylogenetic tree consisting of three elements (chr6 #1, chr7 #1, chr20 #4) of the 53-bp group. Gray bars show expression level.

Following the extraction of repetitive units, we conducted a comprehensive sequence conservation analysis of each element and generated sequence logos (Fig. S5), which revealed the coexistence of conserved and variable sequences. The 5′ and 3′ regions of all D20S16 repeat units are conserved, with the variable region located in the middle of the units (starting from 25 bp). We categorized them into 4 types on the basis of their sequence length: that characterized by a conserved “CATCAG” sequence is 47 bp; that by “CAA-A–G” is 49 bp; that by “CAACACCAG” is 50 bp; and that by “CAGCAGCACC-G” is 53 bp (Fig. 4A).

Specifically, the 47-bp group comprises 7 elements found on chrs. 2, 3, and 5 (1003 units); the 49-bp group comprises 5 elements from chrs. 8 and 20 (1195 units); the 50-bp group comprises 5 elements across chrs. 2, 3, 5, 8, and 9 (339 units); and the 53-bp group comprises 3 elements, including the prominent chr20#4 (272 units). For each of these sequence groups, phylogenetic trees were constructed to further explore their expression in relation to the variable regions.

Our analysis of the phylogenetic tree of the 53-bp group (Fig. 4B) revealed that, with a few exceptions, the units of chr20#4 clustered together, suggesting that this region is a result of tandem repeat duplication that might have increased the unit numbers during evolution.

In addition, we examined the relationship between expression levels and sequence similarity. Intriguingly, despite high sequence homogeneity, expression was exclusive to chr20#4 (gray bars in Fig. 4B). This pattern was also observed in the 50-bp group containing chr20#5 (Fig. S6). These findings prompted us to reassess the mechanisms governing D20S16 expression. From our observations, expression seems to be more intricately associated with specific chromosomal locations than with sequence variation. However, the precise mechanisms and functions warrant further investigation.

### Specific expression of D20S16 in human embryonic development

To broaden our understanding of the role of satellite DNA in embryonic development, particularly D20S16, we explored its expression patterns during macaque embryonic development. We selected rheMac10 (Rhe10) as the reference macaque genome^33^ and used the associated RNA-Seq dataset (SRA ID: SRP089891)^34^ for analysis, which includes single oocytes/embryos or multiple biological replicates at each developmental stage. In addition, it pools samples of 5 to 23 oocytes or embryos collected from 1 to 10 female macaques at each developmental stage.

**Fig. 5:**
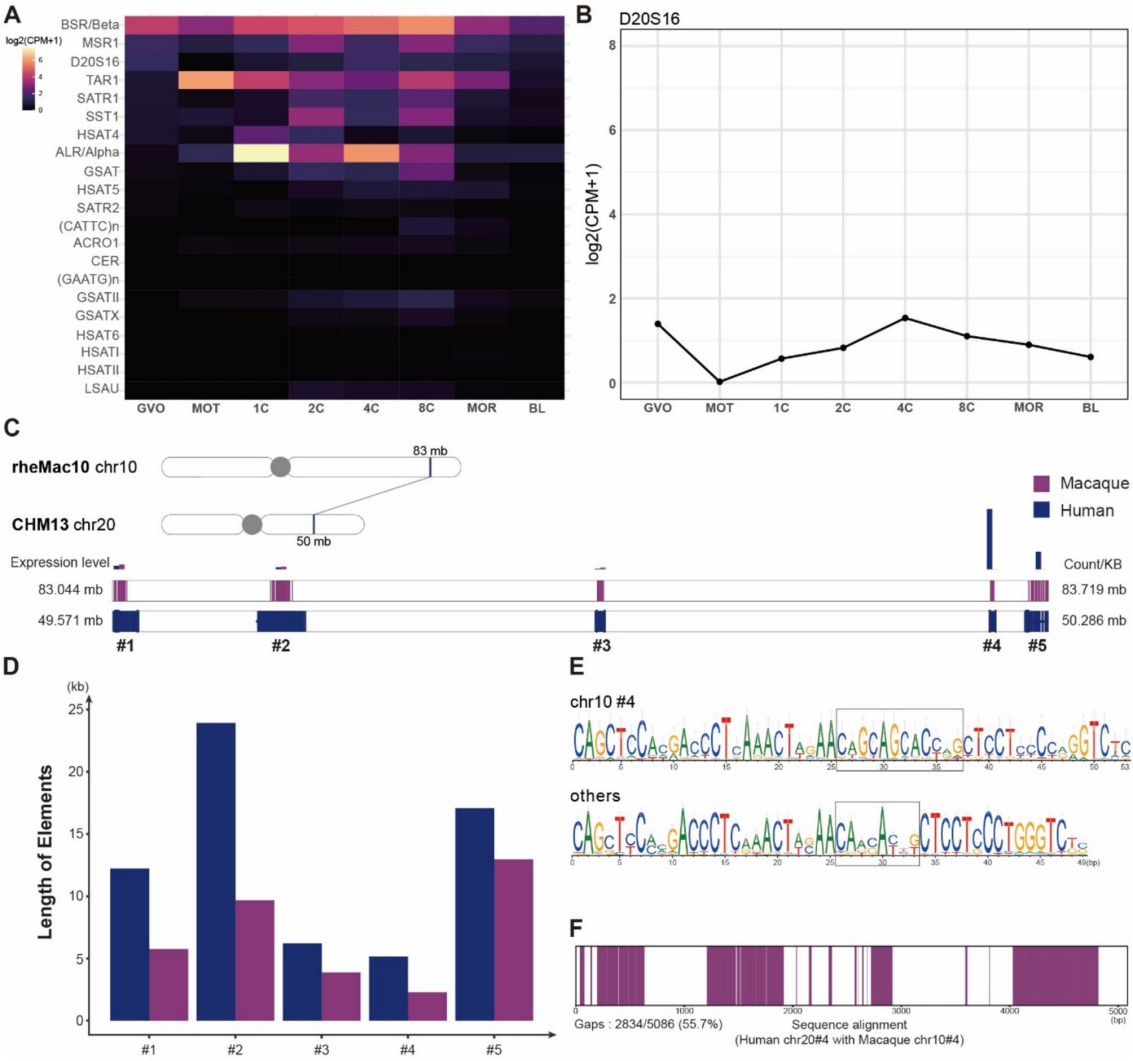
Expression and sequence analysis of D20S16 in macaque embryonic development. (**A**) Heatmap detailing the expression levels of 22 satellite DNA sequences across eight stages of macaque embryonic development: mature blastocyst stage oocytes (GVO), mature oocytes (MOT), 1-cell embryos at the prokaryotic stage (1C), 2-cell embryos (2C), 4-cell embryos (4C), 8-cell embryos (8C), morula (MOR), blastocysts (BL). CPM = counts per million. To visualize gene expression levels, we normalized data as log_2_(CPM + 1). **(B)** The negligible developmental expression pattern of D20S16 during macaque embryogenesis. **(C)** Homologous Regions of D20S16 in Rhe10 (macaque) and CHM13 (human), Sequence Length, and Expression Levels of Various Elements. **(D)** Comparison of sequence lengths of D20S16 in 5 elements between CHM13 and Rhe10. **(E)** Consensus sequence of D20S16 in 5 elements on chr10, and comparison of #4 with other elements. **(F)** Sequence Alignment of CHM13 chr20 #4 with Rhe10 chr10 #4.

We compared the expression patterns of D20S16 between humans and macaques during embryonic development. In contrast to the high expression levels observed in humans, D20S16 expression was barely detectable in macaque early development (Fig. 5A). Even during the cleavage stages (2C-8C), where D20S16 is strongly expressed in humans, its expression remained minimal in macaques (Fig. 5B). This clear difference highlights the pronounced disparity in D20S16 expression between the two species.

To investigate whether these differences in expression are related to variations in the D20S16 sequence, we compared the genomic regions containing D20S16 in humans and macaques. As shown in Fig. 2C, the 50 Mb region of the human chromosome 20 contains five D20S16 elements, of which two were transcriptionally active. By using BLAST^35^, we identified a syntenic region in the macaque genome at 83 Mb on chromosome 10, corresponding to this locus in the human genome (Fig. 5C). We then precisely aligned the five D20S16 elements between the two genome sequences. As a result, we found that the macaque genome also contains five elements; however, in all cases, their lengths are shorter than those in the human elements (Fig. 5D). Notably, the #4 element, which is highly expressed in humans, was only half the length in macaques. This length difference indicates that the number of repetitive units in macaques is lower than in humans, potentially contributing to the observed differences in expression levels.

By applying the same workflow, we extracted the consensus sequence from the corresponding region in Rhe10 and found that the variable region of chr10 #4 shares the sequence ‘CAGCAGCACC-G’ with chr20 #4 in CHM13 (Fig. 5E). Sequence alignment between chr20 #4 in CHM13 and chr10 #4 in Rhe10 revealed gaps (Fig. 5F), suggesting that either humans gained additional repetitive units or macaques lost them during evolution.

### Expression of D20S16 in breast cancer and other human tissues

**Fig. 6:**
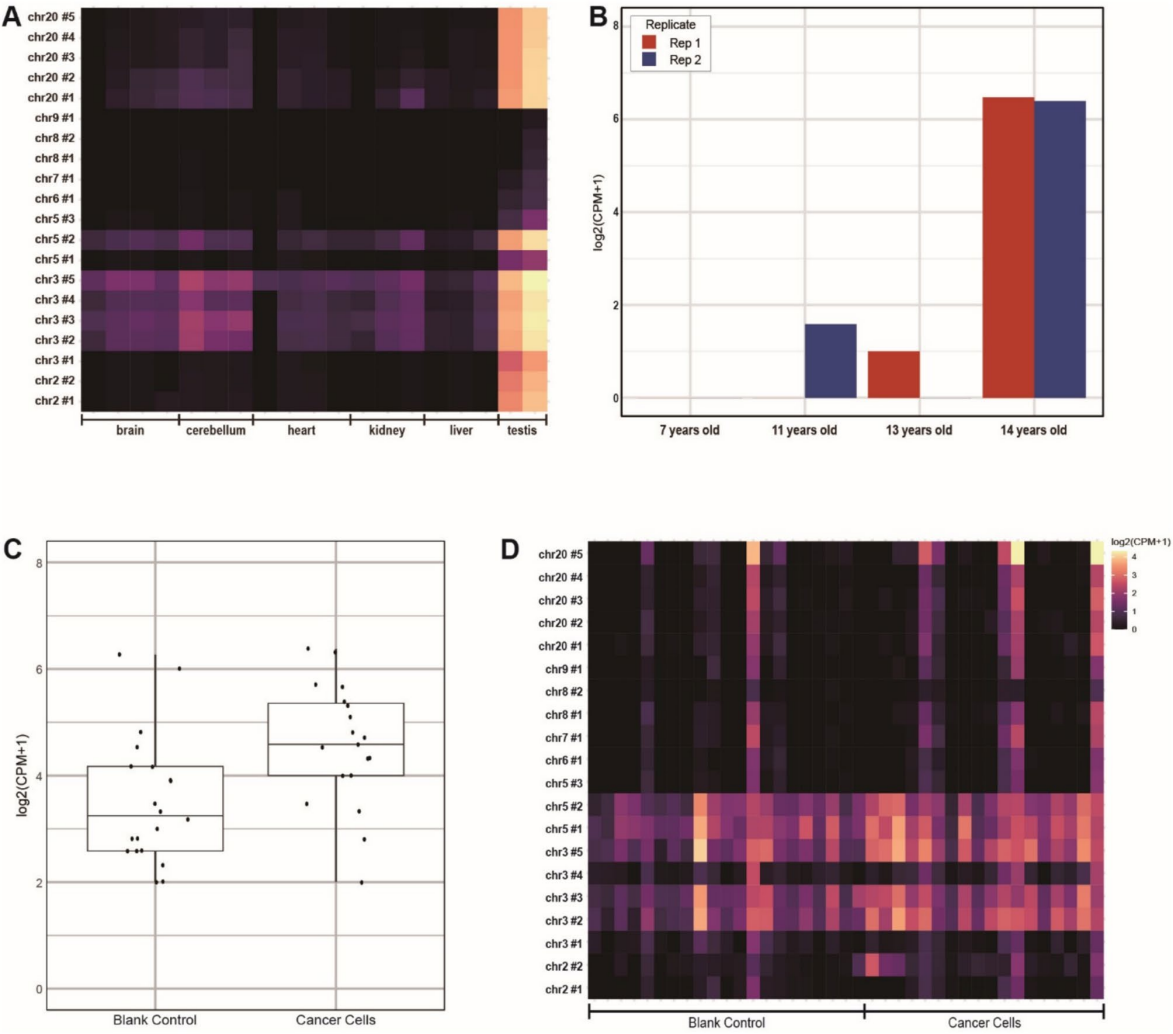
Expression profiles of D20S16 in various human tissues and breast cancer cells. (**A**) Heatmap showing D20S16 expression across six human tissues, with notably higher expression levels observed in the testis relative to brain, cerebellum, heart, kidney, and liver tissues. **(B)** Temporal analysis of D20S16 expression from pre- to post-puberty stages, demonstrating a marked increase at ages 13–14 years. **(C)** Boxplot depicting significantly higher D20S16 expression in breast cancer cells compared with the blank control group (p < 0.01). **(D)** Heatmap illustrating D20S16 expression across individual chromosomal loci in breast cancer cells compared to controls, highlighting predominant expression on chromosomes 3 and 5.

To investigate whether D20S16 is expressed beyond early embryonic stages, we analyzed publicly available RNA-Seq datasets from multiple adult human tissues^36^, including brain, cerebellum, heart, kidney, liver, and testis. Most of these tissues exhibited moderate D20S16 expression; however, testis tissues displayed markedly higher expression levels, comparable to those observed in early embryogenesis (Fig. 6A). To further understand the temporal expression dynamics of D20S16 in testicular development, we analyzed RNA-Seq data from males aged 7, 11, 13, and 14 years^37^, covering crucial developmental stages from pre- to post-puberty. Our results revealed a significant increase in D20S16 expression between ages 13 and 14, suggesting a potential regulatory association with androgen signaling pathways (Fig. 6B). Nevertheless, direct experimental evidence confirming whether D20S16 expression is directly responsive to androgen signals or functionally related to germ cell differentiation remains lacking, rendering this hypothesis speculative at present.

Given our findings suggesting a possible association between D20S16 expression and androgen signaling during testicular development, we next hypothesized whether estrogen signaling might be similarly associated with D20S16 expression. To address this, we analyzed RNA-Seq data from 22 estrogen receptor-positive breast cancer samples and their paired adjacent non-malignant tissues^38^. Our analysis demonstrated significantly elevated expression of D20S16 in breast cancer tissues compared to matched controls (Student’s t-test, p-value = 0.0087; Fig. 6C). Notably, while D20S16 expression during embryogenesis was restricted to specific loci on chromosome 20, breast cancer tissues showed predominant activation at previously inactive regions on chromosomes 3 (#2, #3, #5) and 5 (#1, #2) (Fig. 6D). Further inspection revealed that these newly activated regions were primarily localized around telomeric areas. These observations suggest a potential role of D20S16 in telomere maintenance mechanisms within cancer cells^39^.

### Comparative genomics analysis of D20S16

**Fig. 7:**
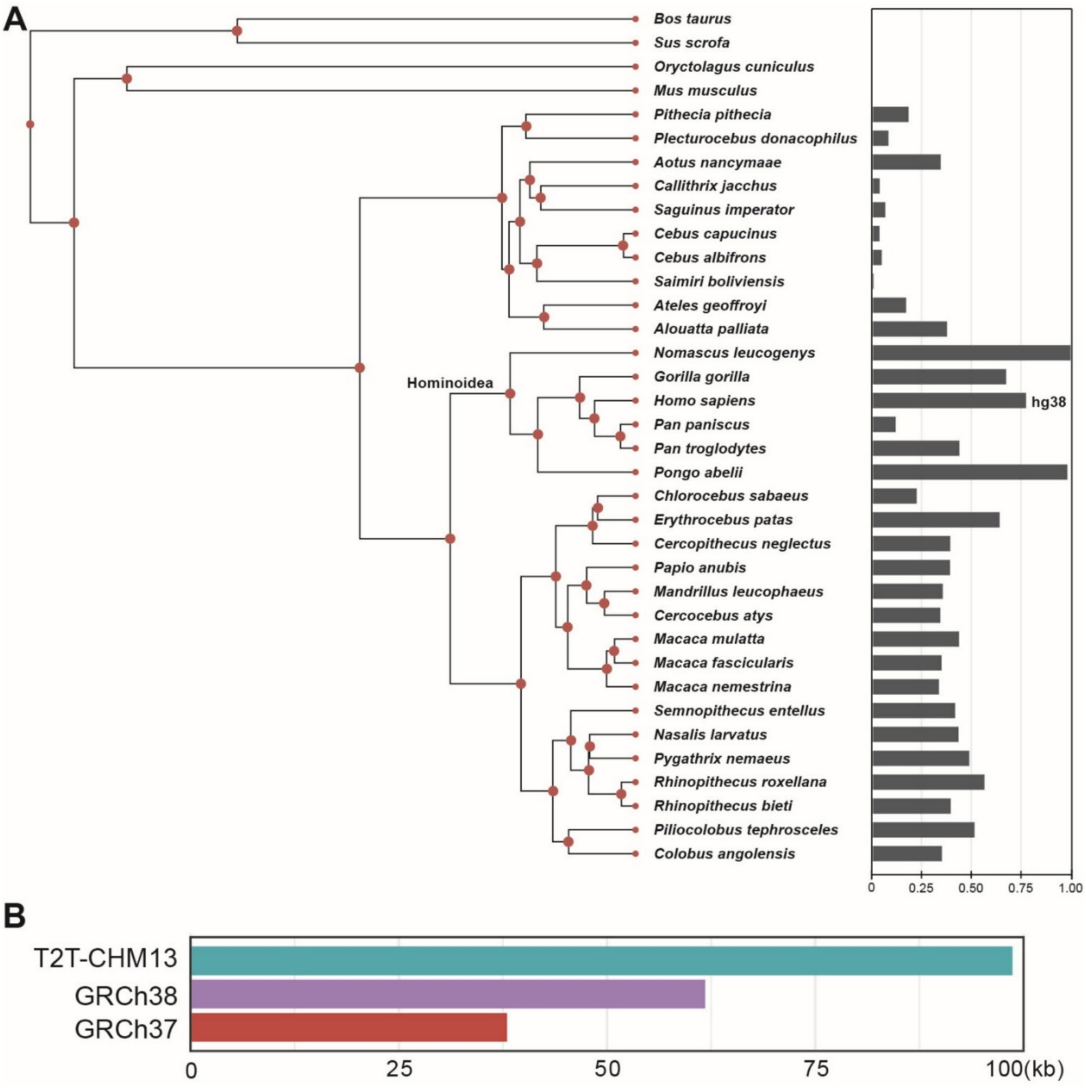
Evolutionary distribution and regional variability of D20S16 satellite DNA sequences. (**A**) Phylogenetic tree illustrating evolutionary relationships among selected primates and representative mammals (cattle, pig, rabbit, and mouse) based on Dfam genomic database analysis. Horizontal bars indicate normalized lengths of the D20S16 satellite DNA sequence. **(B)** Bar chart comparing the annotated lengths of the D20S16 region across three human genome assemblies: GRCh37 (hg19), GRCh38 (hg38), and the complete T2T-CHM13 assembly.

To explore the evolutionary distribution of the satellite DNA sequence D20S16, we first analyzed its presence in primates and four other representative mammalian species—cattle *(Bos taurus)*, pig *(Sus scrofa)*, rabbit *(Oryctolagus cuniculus)*, and mouse *(Mus musculus)*. Using repeat annotations generated by the Dfam database—which applies Hidden Markov Models (HMMs) to identify repetitive elements—we extracted and quantified the total lengths of D20S16 across multiple available genomes. The resulting data—representing total D20S16 lengths detected per genome—were normalized relative to the cumulative D20S16 length across all analyzed species, and used to construct a phylogenetic tree representing evolutionary relationships. (Fig. 7A). Our results indicated that members of the Hominoidea superfamily generally possess longer D20S16 sequences compared to other primates. Notably, the Northern white-cheeked gibbon *(Nomascus leucogenys)* and Sumatran orangutan *(Pongo abelii)* exhibited the longest D20S16 sequences, whereas bonobo *(Pan paniscus)* presented substantially shorter sequences. Although humans *(Homo sapiens)* ranked third in D20S16 length, it is important to emphasize that the human data used here are derived from the hg38 reference genome rather than the more complete T2T-CHM13 assembly, suggesting the actual D20S16 length in humans may be underestimated. Additionally, no signal for D20S16 was detected in cattle, pig, rabbit, or mouse genomes according to the Dfam database, a finding we subsequently confirmed using BLAST analyses. It is noteworthy that the absence of detected D20S16 sequences in some primate species could also be due to the limited genome data available in the Dfam database.

In the second part of this analysis, we focused on comparing the annotation of the D20S16 region across three human reference genome assemblies: GRCh37 (hg19), GRCh38 (hg38), and the complete T2T-CHM13 assembly (Fig. 7B). Our analysis clearly showed that the D20S16 sequence in T2T-CHM13 is substantially longer than in hg19 and hg38. This result suggests that earlier assemblies likely underestimated the repeat content due to technical limitations in resolving highly repetitive regions. We interpret the shorter lengths observed in hg38 and hg19 not as evidence of true structural variation, but rather as a reflection of incomplete assembly in these older references. These findings emphasize the importance of using complete, telomere-to-telomere assemblies for accurate characterization of satellite DNA regions.

## Discussion

This study explored the expression of satellite DNAs throughout human embryonic development, providing insights into potential regulatory roles during embryonic development. Notably, the behaviour of D20S16, GSATII, and TAR1 may indicate that they play significant roles in early developmental stages, possibly orchestrating key transitions and gene expression profiles. Similarly, the observed expression patterns of ACRO1 and BSR/Beta might align with their potential roles in critical phases of embryonic development, such as cell division and differentiation processes. The transient expression peaks of ALR/Alpha and (GAATG)n could be indicative of their importance during specific windows of embryonic development, although the direct correlation to specific biological functions remains speculative.

Among these, D20S16 emerged as particularly noteworthy owing to its pronounced expression levels and distinct trend, driving a comprehensive investigation into its copy number, chromosomal positioning, and consensus sequences. This analysis enhanced our understanding of satellite DNA and introduced a new perspective: that chromosomal positioning might contribute to the regulatory expression of satellite DNA. Moreover, the elevated expression of D20S16 in pathological contexts, such as breast cancer^40^, highlights its broader biological relevance and potential as a target for therapeutic strategies.

Although our study did not directly assess whether satellite DNA transcription in cancer stems from global transcriptional deregulation, existing evidence suggests a mechanistic link. In various malignancies, epigenetic instability and heterochromatin relaxation frequently accompany hypomethylation and altered chromatin states, events known to permit the aberrant activation of repetitive elements including satellites, LINEs and Alus^41–43^. Therefore, we hypothesize that the D20S16 transcription we observe in ER⁺/HER2⁻ breast tumors may reflect a broader deregulation of silenced genomic elements. It also raises the possibility that additional satellite families might similarly become transcriptionally permissive under oncogenic stress, a hypothesis that warrants comparative transcriptomic analysis across diverse cancer types.

Another important interpretative limitation involves distinguishing maternal and zygotic transcripts. Studies in mice and humans have shown that maternal mRNA degradation initiates during oocyte maturation and proceeds rapidly following fertilization, with most transcripts being cleared by the 2- to 4-cell stage as zygotic genome activation begin^44^. In our dataset, however, we did not observe a notable decrease in D20S16 expression after the MII oocyte. On the contrary, expression either remained stable or increased, suggesting that the signal is unlikely to be derived from maternally stored RNA. Nonetheless, due to the lack of direct experimental evidence, we acknowledge this as an interpretative limitation. Future studies should consider applying metabolic RNA labelling or allele-specific expression strategies to determine transcript origin.

While previous studies^24^ focused on expression analysis, our research also examined the genomic regions of individual elements where this expression occurs. By using a combination of manual curation combined with HMM-based identification, we constructed HMM profiles for D20S16 subtypes. This allowed us to identify the variable and conserved regions within D20S16. Although this lays a solid foundation for further research, we must acknowledge that the subjectivity inherent in manual extraction and the assumptions underlying the HMM may introduce unconfirmed variations.

Notably, our study did not find a D20S16 expression pattern in the macaque model analogous to that in humans. While this reinforces the hypothesis that D20S16 exhibits a unique expression pattern in human embryonic development, it also reflects current limitations in the available primate data. Although the macaque genome used in our analysis may not fully represent the repetitive regions, recent advances such as the single-cell transcriptomic datasets from early monkey development provided by Oomen et al. (2024)^45^ offer valuable opportunities for further exploration. Future research should incorporate such high-resolution datasets to better resolve the evolutionary and functional divergence of satellite DNA expression across primates.

Future research should focus on the experimental validation of the function of D20S16, mainly through the precise manipulation of its expression in model organisms by using gene editing technologies, such as the CRISPR-Cas9 system. This could reveal its specific effect on embryonic development. If feasible, creating transgenic mice carrying the human D20S16 sequence through gene knock-in experiments could provide insights into its role throughout embryonic development. In addition, although RNA-Seq provides comprehensive transcriptome-wide data, it may have limitations in accurately quantifying highly repetitive sequences such as satellite DNA. Therefore, real-time quantitative PCR (qRT-PCR) could serve as a complementary approach, offering precise quantification of D20S16 expression at specific developmental stages. This method could validate RNA-Seq findings and help detect expression differences in experimentally challenging or underrepresented stages. Future studies should investigate the interactions between D20S16, other genes, and signalling pathways during embryogenesis, as well as its specific roles in cell division and differentiation.

In summary, our study offers new insights into the expression patterns of D20S16 in human embryonic development. These findings could pave the way for new fields of research into the function of satellite DNA and potentially provide new understandings and therapeutic strategies for diseases related to embryonic development.

## Methods

### Collecting transcriptome and genome data

We used the fasterq-dump v. 3.0.3 tool to retrieve RNA-sequencing (RNA-Seq) data derived from 658 cells and 667 developing human embryos (GEO Accession GSE85632) in FASTQ file format from the Sequence Read Archive (SRA) database, and to retrieve RNA-Seq data of human (GSE71318) and macaque (GSE86938) supplementary cells of developing embryos. We used the T2T-CHM13 v. 1.1 human genome reference and the rhcMac10 macaque genome reference and their corresponding gene annotations in GTF format from the UCSC Genome Browser (https://genome.ucsc.edu/). Sequence information for satellite DNA was sourced from the RepeatMasker database (https://www.repeatmasker.org/).

### Profiling expression of satellite DNA

For read mapping, we first pre-processed the data in FastQC v. 0.11.9 to perform quality analysis with the command ‘*fastqc -t 2 -q*’. We then used trim_galore v. 0.6.7 (‘*trim_galore -q 20 –nextera –paired*’) to remove base sequences with quality scores < 20 and to trim Nextera primers. To remove rRNA reads, we processed the dataset in rRNAdust v. 1.06. The paired fastq files were then concatenated in seqkit^46^ v. 2.2.0 (‘*seqkit pair −1 file1 −2 file2*’). Index generation and read mapping were conducted in STAR^47^ v. 2.7.10a. The resulting read mappings were then normalized to the volume of RNA-Seq data in the dataset, and counts per million (CPM) values were computed across all samples. Finally, to visually represent the expression of satellite DNA during embryonic development, heatmaps and line graphs were generated and presented in R software.

### Merging D20S16 copies

Owing to the sequence similarity between MLT2B4 and D20S16, which complicates the identification of D20S16 sequences, we merged copy regions that were separated because of this similarity. First, we measured the distance between adjacent copies on each chromosome, using *regular expression*. This process entailed calculating the distance by subtracting the end position of a given copy from the start position of the next copy on the same chromosome. Typically, the distances between proximate copies ranged from 10 to 200 bp. Copies that were separated by more than 1000 bp were considered sufficiently distant to be classified as distinct elements. We used Mafft^48^ v. 7.515 for comprehensive sequence comparison and further examination of these regions, and visualized the results in the Integrative Genomics Viewer^29^.

### Extraction of repeat unit in D20S16

Upon acquiring the data from the merged region, we initiated extraction by using a Regex command in *awk* to isolate the conserved region “CAGCT” as the reference locus. Then we used Mafft for multi-sequence alignment (*mafft –thread 12 –globalpair –maxiterate 1000*) of the segmented regions to scrutinize the initial cutting results. During this phase, any inaccuracies due to non-conservative sequences were identified and rectified. This cycle of cutting, aligning, and correcting was performed two or three times to enhance the precision of the extracted sequences. Through these iterations, a more comprehensive multiple sequence comparison file was compiled. Following this, HMMER^49^ v. 3.3.2 was used to construct a model (hmmbuild) and search for sequences similar to the identified unit across the entire genome (hmmsearch). Finally, to visually represent the consensus sequence, we generated sequence logos in weblogo^50^, using custom Python scripts. This graphical representation provides a clear and concise visualization of the sequence conservation and variability within the repetitive unit of D20S16.

### Phylogenetic tree construction

The consensus sequence was generated from all units in Mafft for multiple sequence comparison. The specific sequences spanning positions 26–37 within the consensus sequence were precisely extracted through the use of custom Python scripts. This targeted approach allowed for a focused analysis of the region of interest. Thereafter, the extracted sequences were used to construct a phylogenetic tree in PhyML^51^ v. 3.3.20220408.

### Processing of single cell RNA-seq data

Raw data were demultiplexed using the mkfastq application (Cell Ranger v9.0.1) to generate Fastq files. These Fastq files were then processed using the count application (Cell Ranger v9.0.1) with default settings, which includes alignment via the STAR aligner. The resulting read mappings were subsequently normalized based on the total RNA-Seq data volume, and counts per million (CPM) values were calculated for all samples.

## Supplementary Information

Supplementary Information.

## Data Availability

The datasets analysed during the current study are available in the GEO repository. The primary analysis of satellite DNA expression during human embryonic development was conducted using dataset GSE72379, with supplementary insights provided by GSE71318. Additionally, dataset GSE86938 was employed to study the expression of satellite DNA during macaque embryonic development. Dataset GSE134144 was utilized to examine D20S16 expression during testicular development across puberty, while dataset GSE30352 provided RNA-seq data for comparative analysis across multiple human tissues. Furthermore, breast cancer expression analysis was performed using dataset GSE103001, which contains RNA-seq data from estrogen receptor-positive tumors and adjacent non-malignant tissues.

## Abbreviations

RNA-Seq: RNA sequencing
HMM: Hidden markov model
CPM: Counts per million
GRCh38: Genome reference consortium human build 38
T2T-CHM13: Telomere-to-telomere CHM13 (Complete human genome)
IGV: Integrative genomics viewer
LTR: Long terminal repeat
ERV: Endogenous retrovirus
GTF: Gene transfer format
FASTQ: FASTQ (Sequence file format)
SRA: Sequence read archive
HMMER: Hidden markov model software
qRT-PCR: Quantitative real-time polymerase chain reaction
CRISPR-Cas9: Gene editing technology
BLAST: Basic local alignment search tool
