# Supplementary material for: Unveiling unique expression patterns of D20S16 satellite DNA in human embryonic development
Source: Sci Rep. 2025 Jul 23;15:26770. doi: 10.1038/s41598-025-11753-w (PMC12287470; doi:10.1038/s41598-025-11753-w)
Supplement: Supplementary file 1 — Supplementary Information. [file 41598_2025_11753_MOESM1_ESM.pdf]

Supplement Figures and Table

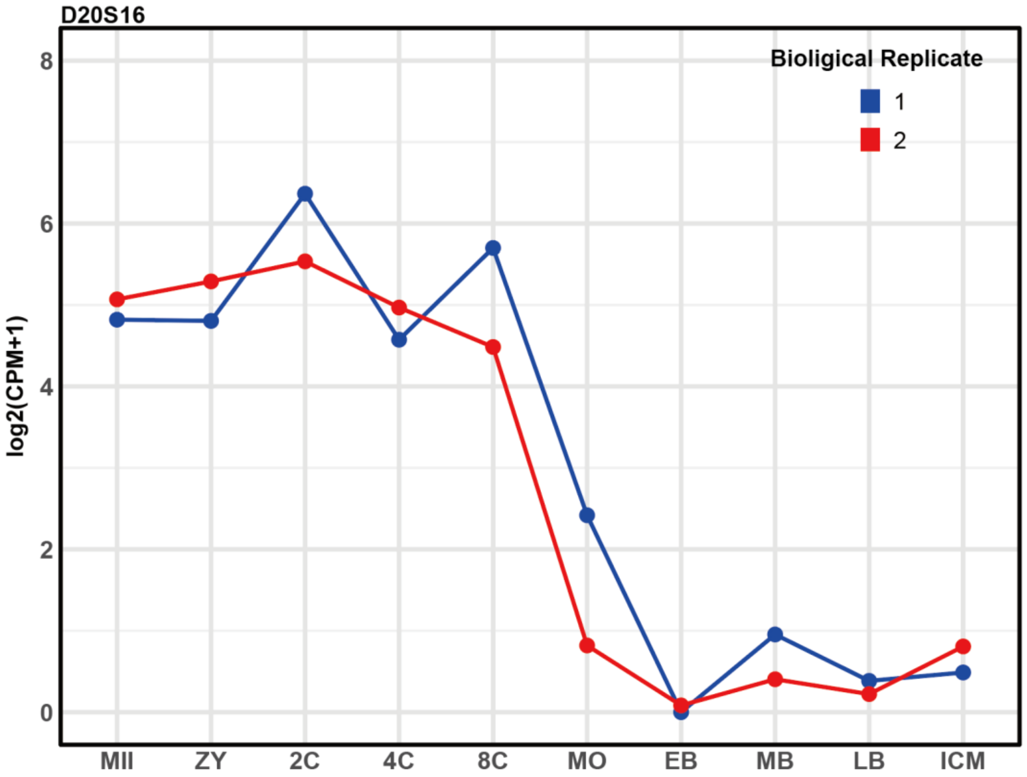

Figure S1 Expression pattern of D20S16 across various developmental stages: MII oocyte (MII), zygote (ZY), 2-cell (2C), 4-cell (4C), 8-cell (8C), morula (MO), early blastocyst (EB), middle blastocyst (MB), late blastocyst (LB), to ICM (Inner Cell Mass) with 2 biological replicates.

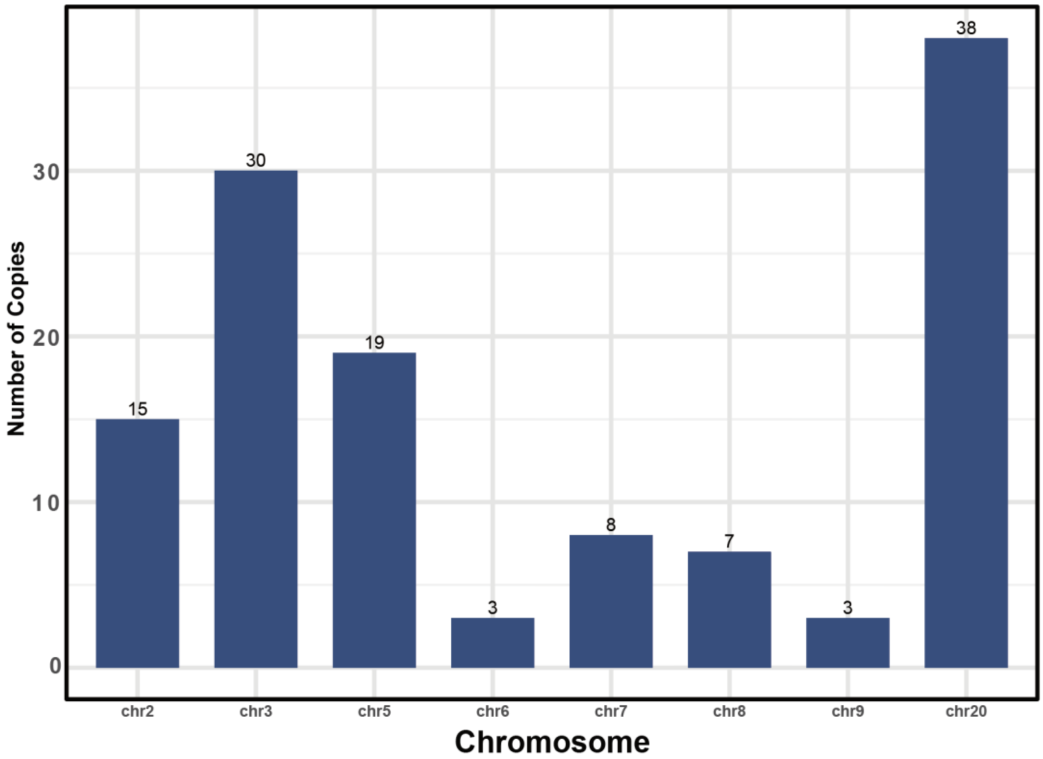

Figure S2 Bar graph showing the count of D20S16 copies distributed across different chromosomes. There are 38 copies on chr. 20 and 30 on chr. 3.

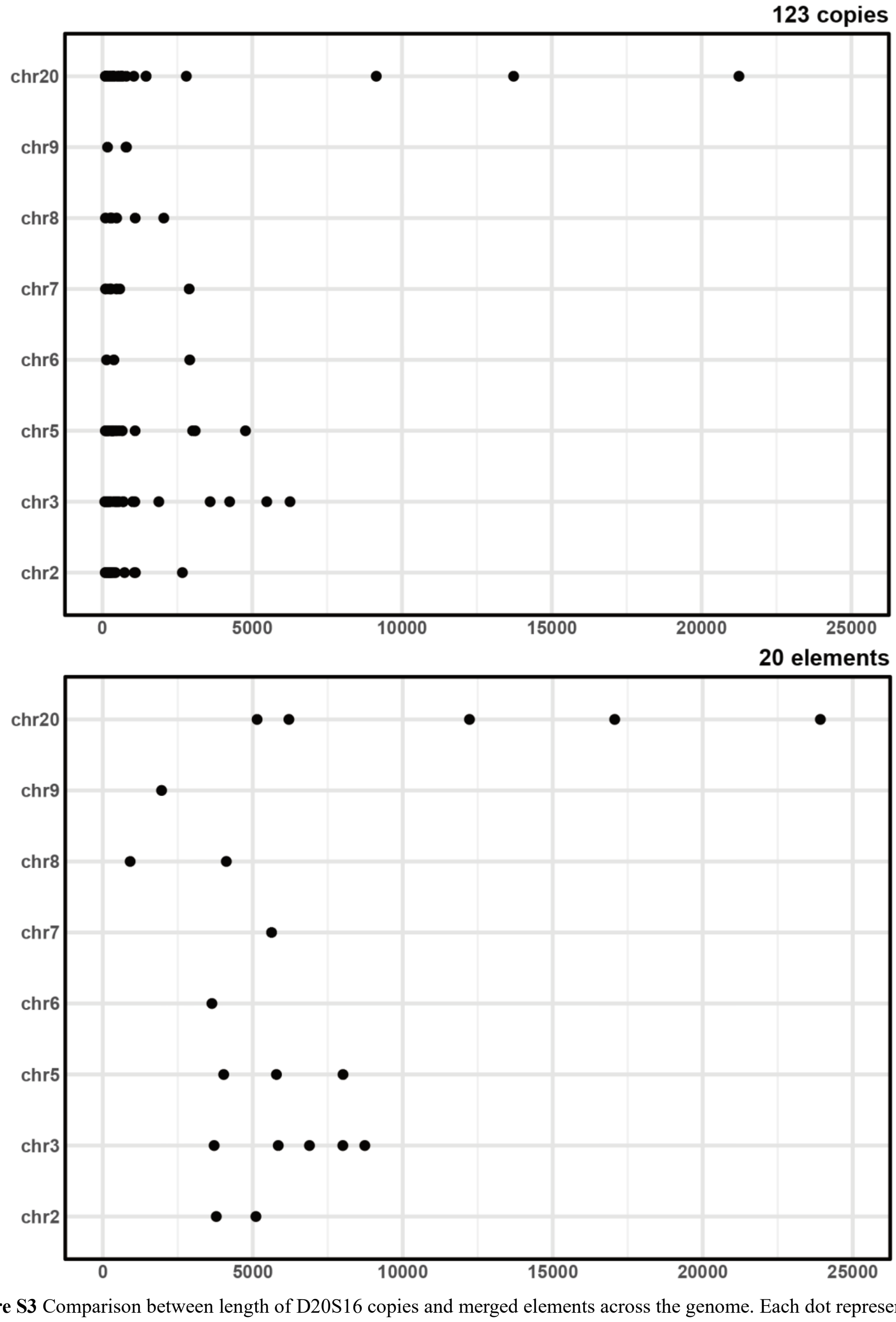

Figure S3 Comparison between length of D20S16 copies and merged elements across the genome. Each dot represents the length of a copy (element). The numerous short copies before merging become larger elements after merging.

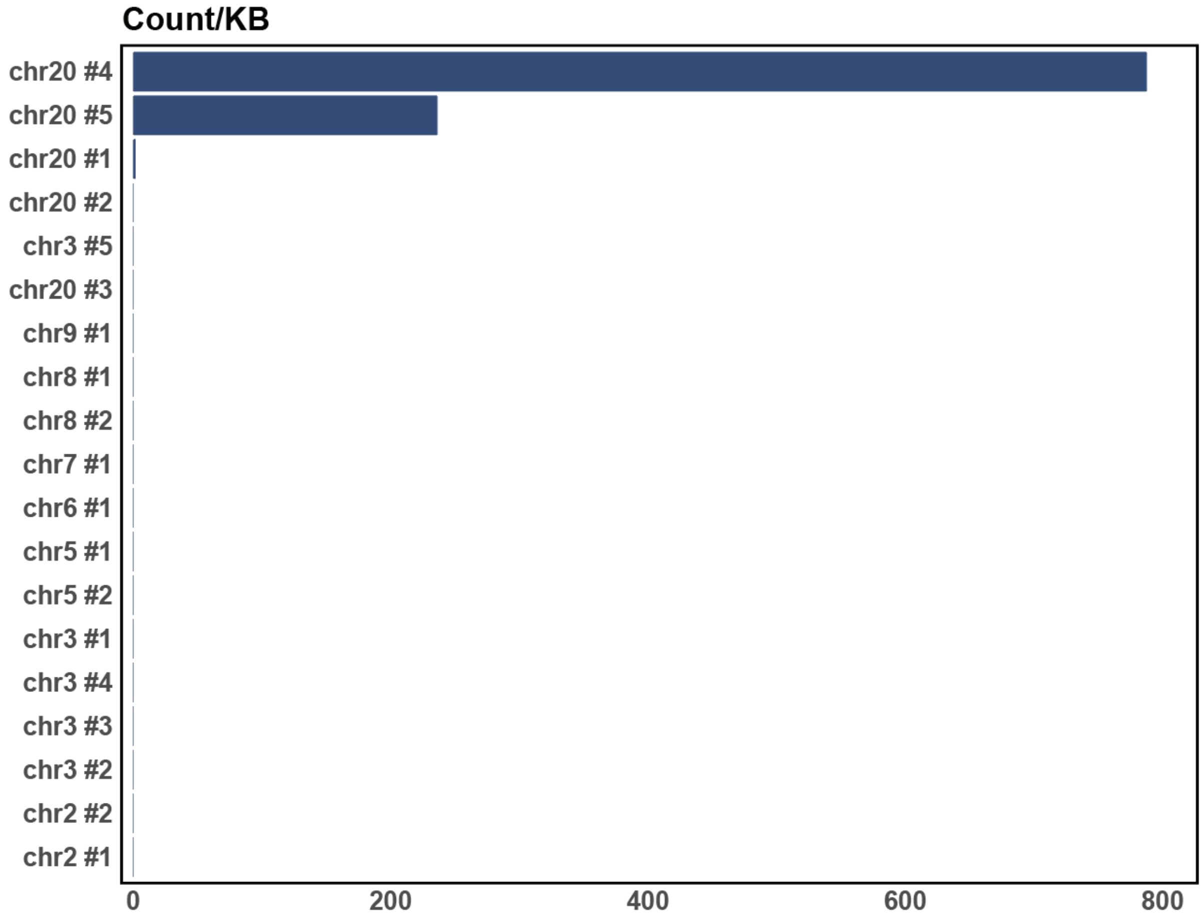

Figure S4 Relative expression levels of D20S16 elements across different chromosomes in the extended dataset. Expression is much higher from chr20#4 and #5 than from the other elements.

Element	Start	End	strand
Chr2#1	188355151	188358936	+
Chr2#2	242530792	242535898	-
Chr3#1	849648	853361	+
Chr3#2	198351002	198359006	-
Chr3#3	198412650	198418500	-
Chr3#4	198718124	198725015	+
Chr3#5	200369180	200377916	+
Chr5#1	271481	277271	-
Chr5#2	1531375	1539385	+
Chr5#3	26328676	26332708	+
Chr6#1	151251605	151255244	+
Chr7#1	57459439	57465067	-
Chr8#1	69751737	69755856	-
Chr8#2	125141718	125142632	-
Chr9#1	149010311	149012272	+
Chr20#1	49571527	49583752	+
Chr20#2	49643235	49667158	-
Chr20#3	49889301	49895506	+
Chr20#4	50241412	50246557	+
Chr20#5	50269209	50286276	+

Table. S1 Names, starting points, ending points, and strands of the 20 elements from D20S16.

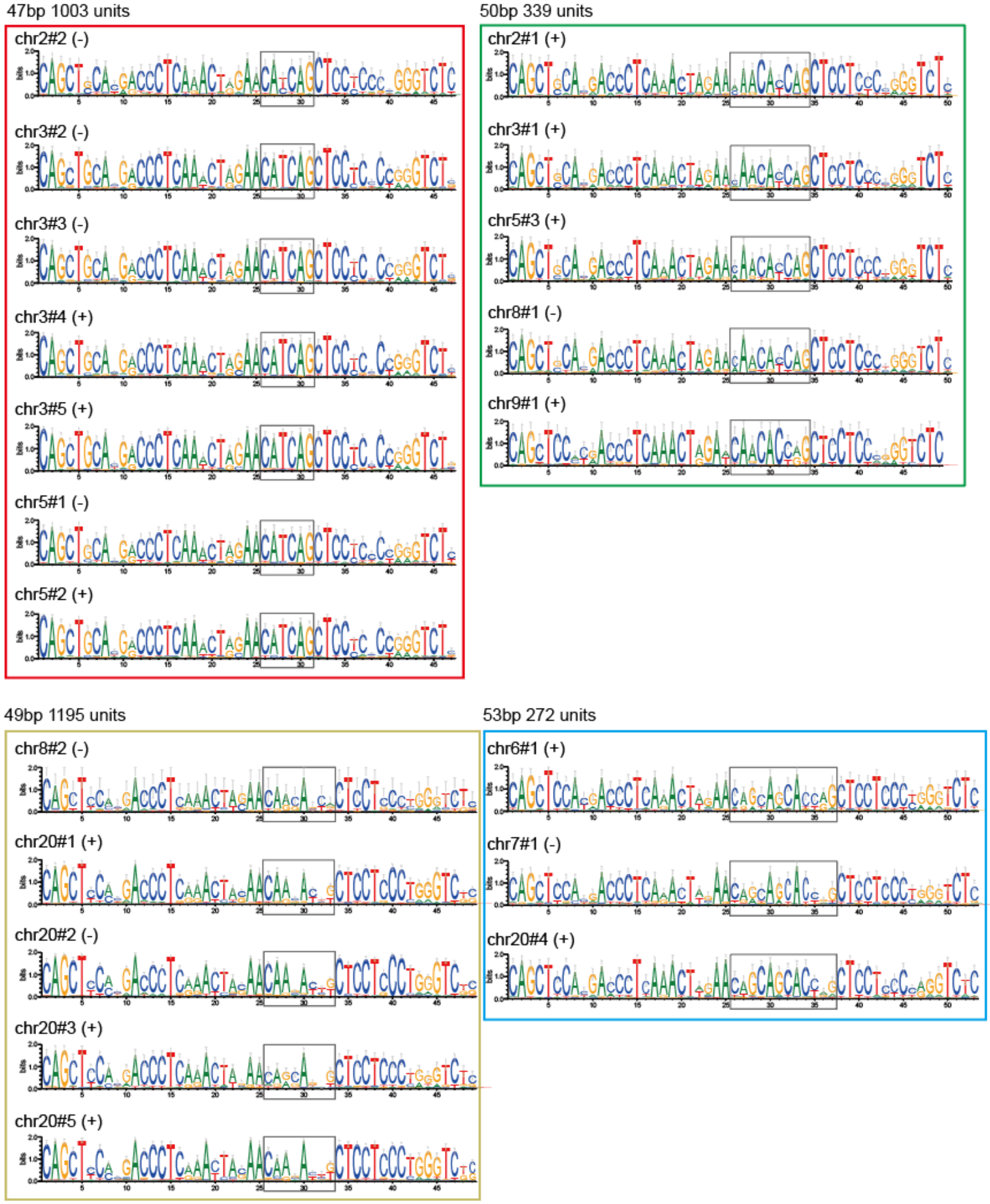

Figure S5 Consensus sequences and variable regions of 4 types of D20S16 (47, 49, 50, 53 bp). Sequence logos are used to highlight the nucleotide diversity at each position. The 47-bp type (CATCAG) has 1003 units, found on chrs. 2, 3, and 5. The 49-bp type (CAA-A--G) has 1195 units, found mainly on chrs. 8 and 20. The 50-bp type (CAACACCAG) is more dispersed, with 339 units found on chrs. 2, 3, 5, 8, and 9. The 53-bp (CAGCAGCACC-G) group has 272 units, including chr20#4.

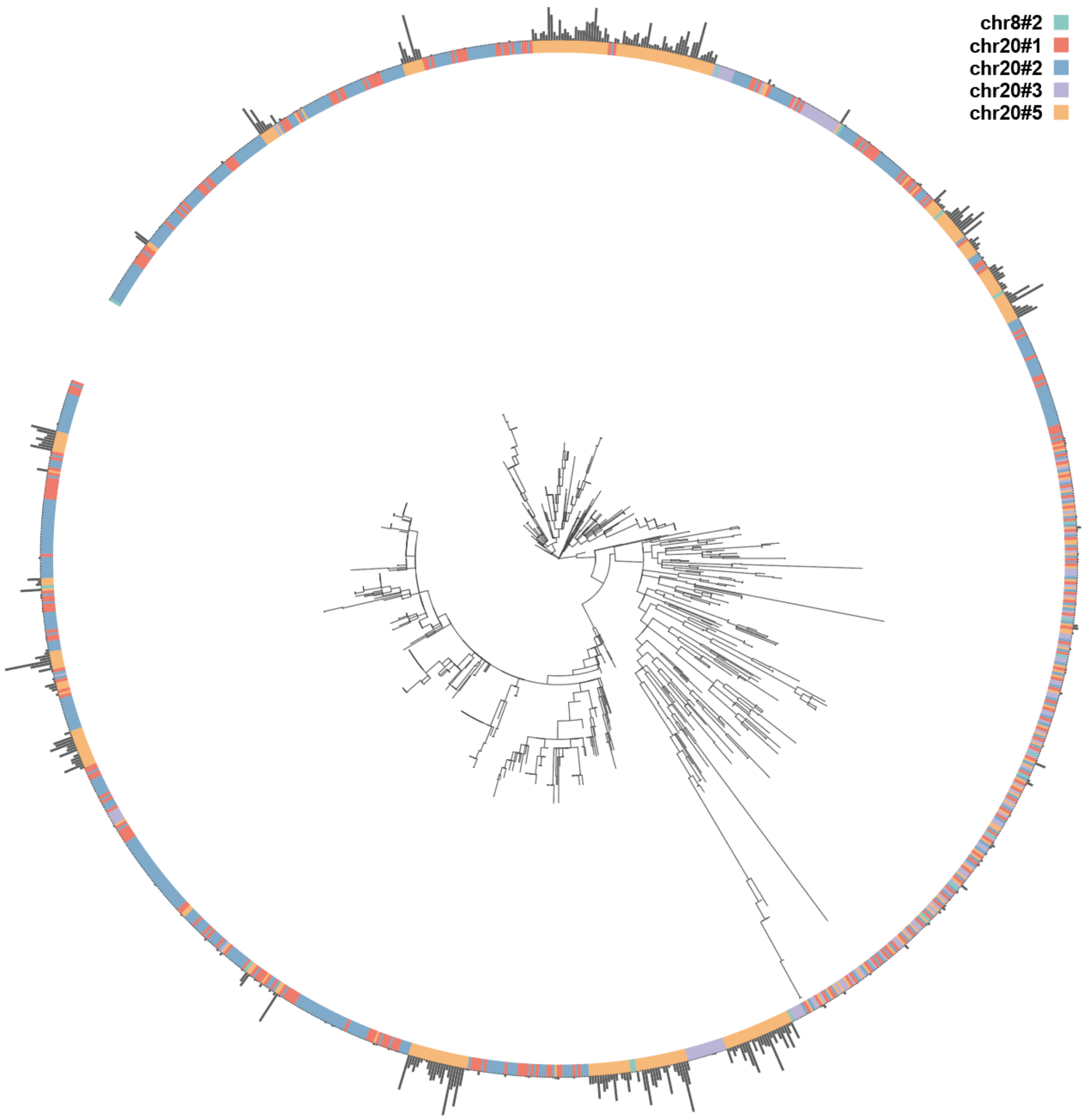

Figure. S6 Phylogenetic tree consisting of 5 elements (chr8#2, chr20#1, chr20#2, chr20#3, chr20#5) of the 49-bp group, with gray bars showing expression level.
